# Influence of Comprehensive Life Style Intervention in Patients of CHD

**DOI:** 10.5539/gjhs.v7n7p6

**Published:** 2015-03-26

**Authors:** Ali Dehghani, Sanjiv Kumar Bhasin, Shridhar Dwivedi, Rajeev Kumar Malhotra

**Affiliations:** 1 Epidemiology Department, Health College, Shaheed Sadoughi Medical University, Yazd, Iran; 2 Department of Community Medicine, UCMS &GTB Hospital, University of Delhi, India; 3 Department of Medicine/Preventive Cardiology HIMSR & HAH, Centenary Hospital, Hamdard University, Delhi, India; 4 Department of Biostatistics and Medical Informatics, UCMS &GTB Hospital, University of Delhi, India

**Keywords:** Coronary heart disease, Lifestyle intervention, Risk factors

## Abstract

**Background and Objectives:**

Over the past 30 years, the CHD rates have doubled in India whereas CHD rates have declined by 15% in most developed countries due to lifestyle interventions during the same period. So, the present study was conducted to find out the effectiveness of lifestyle intervention in reducing major risk factors in CHD patients in an Indian setting.

**Methods:**

We conducted this randomized controlled trial on 640 eligible subjects who were randomly assigned to two groups. The study group was given an interventional package at baseline and at three months, detailing the aspects of a healthy lifestyle in relation to CHD risk factors whereas no intervention was provided for the control group. The study subjects were followed at three and six months and the risk factors were assessed to find out reduction, if any, in the prevalence of the risk factors amongst them.

**Results:**

There was a significant reduction in hypertension, tobacco, and lack of physical activity at three and at six months (p<0.03) when compared to the baseline in the study group. However, there was no significant reduction in obesity at three months (p=0.148) while the reduction in obesity was significant at six months (p=0.0005) in the study group as compared to the control group. The lipid profile reduced significantly at six months but there was no statistically significant reduction in diabetes at six months in the study group as compared to the control group (p=0.419).

**Interpretation & Conclusion:**

Except for diabetes, the lifestyle intervention was successful in increasing physical activity, improving the hypertension control, and decreasing lipid profile disorders, obesity, and tobacco use in the study group.

## 1. Introduction

Cardiovascular diseases, especially coronary heart disease (Lopez, Mathers, Ezzati, Jamison, & Murray, 2006), are the leading cause of mortality in the developing world. The CHD prevalence in South Asia is 3.2% (Sachar, 1998). The prevalence is 10% in New Delhi and 11% in Chennai (Mohan, Deepa, Rani, & Premalatha, 2001). This alarming rate signifies the importance of the need for prompt and effective preventive health measures. Different studies have reported that the prevalence of the risk factors of ischemic heart disease in Delhi are as follows: smoking 59% (Dwivedi, Anupam, & Chaturvedi, 1997), obesity 41% (Aggarwal, Chaturvedi, Bhasin, & Gupta, 2000), hypertension 22% (Das & Ashida, 2007), positive family history 17% (Dwivedi et al., 1997), diabetes 10% (Aggarwal et al., 2000), and hypercholesterolemia 8% (Dwivedi et al., 1997).

A decade ago, lifestyle modification (LM) was introduced as an alternative treatment for CHD in order to reduce mortality and improve the quality of life of the patients. Although the studies related to lifestyle modifications have been carried out mainly in the developed countries, few studies have been conducted in India. For this reason, the present study was conducted to find out the effectiveness of lifestyle interventions in reducing the major risk factors in CHD patients admitted to the Coronary Care Unit ward of Gru Teg Bhadur Hospital, Delhi, in a comprehensive manner.

## 2. Material & Methods

Study design and recruitment: It was a randomized controlled trial parallel group study with equal randomization, i.e 1:1, for the study and control groups. The study was conducted from January 2008 to April 2012 at the Coronary Care Unit and Preventive Cardiology Clinic (PCC) of the University College of Medical Sciences and GTB Hospital established in Dilshad Garden area catering the health care needs of East Delhi and adjoining western UP population.

### 2.1 Subjects

Eligibility: The study subjects were male and female consecutive newly diagnosed cases of CHD aged 25-65 years. CHD patients were diagnosed according to Monica criteria: 1) two or more ECGs showing specific changes 2) ECG showing probable changes plus abnormal cardiac injury enzymes or 3) typical symptoms such as retrosternal pain plus abnormal enzymes (Palomaki et al., 1994). The cases were confirmed by an expert cardiologist working in the CCU/Intermediate CCU (ICCU) ward of GTB Hospital and subsequently in the Preventive Cardiology Clinic. Old CHD patients, pregnant women, and patients suffering from dementia and/or severe psychiatric illness were excluded.

Sample size: The event rate among controls, i.e the prevalence of risk factors after six months of coronary heart disease episode following standard treatment, was reviewed from the literature, and an event rate of 20% was found for tobacco use. With an Odds Ratio of 0.5 for two-sided 5% significance level and a power of 90%, the sample size was calculated to be largest for tobacco use with 261 patients. Taking into account the 9% attrition and 9% mortality rate, the final sample size was 318 participants. Therefore, study and control groups each included 320 subjects.

**Figure 1:**
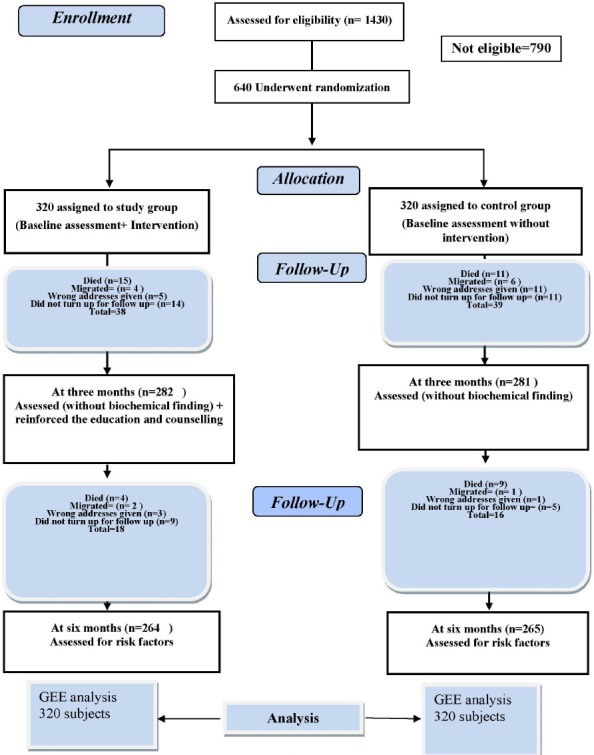
Flow diagram of RCT of lifestyle intervention in the present study

Randomization and allocation concealment: Eligible patients were randomly allocated to the study group or the control group (1:1), using a computer-generated list of random numbers and the sealed-envelope technique. In sealed-envelope technique numbers 1-640 were written on the top of the back of opaque envelopes sequentially. Based on randomization done by random number tables, a small piece of paper having either study or control group written on it with a light pencil was put inside the envelopes and sealed. These envelopes were in the custody of one of the authors (SKB) and were given sequentially every day to the translator who was nurse educator who was equally fluent in Hindi and English. Every envelope was opened after the patient’s admission and performa filling by the first author. Then patients were allocated to the group mentioned on the slip of paper inside envelope. The process of randomization stopped on reaching 640 subjects (320 in the study and 320 in the control group) (Figure 1). No blinding could be done because of the study design.

Intervention: The study group received a package of lifestyle intervention every three months, i.e. once at the time of admission and once at three months to reinforce and modify the intervention message based on the prevailing profile of the patients at the time of counseling. Besides, counseling was reinforced every month when the patients attended the Preventive Cardiology Clinic for follow-up. The intervention team consisted of an investigator and a nurse educator (translator). The intervention was in the form of “one to one basis” respecting the participants’ privacy. After each intervention, there was an interactive session with the patients to fulfill their counseling needs. They were motivated to ask questions and clarify their doubts.

The comprehensive lifestyle interventional package was administered using a power point presentation detailing the aspects of a healthy lifestyle in reducing the risk factors related to CHD i.e. 1) tobacco 2) diabetes mellitus 3) obesity 4) hypertension 5) physical activity and 6) lipid profile. The power point presentation was based on scientifically valid, objective and practical information covering all aspects of CHD with the aim of helping the patients to reduce risk factors. The presentation lasted for about 20 minutes for each patient and was in English or Hindi based on the patient’s knowledge of the languages. The companions of the patients (in the study group) also benefited from the power point presentation. In addition, a small booklet prepared by the Investigating team was given to all patients in the study group. The interventional package was not given to the control group.

An intervention diary was maintained to monitor the degree of compliance by the investigator, the nurse educator (translator), and also all patients.

At the time of CHD diagnosis, six risk factors were assessed in both the study and control groups. Patients in the Control group were managed as per the usual routine, i.e prescribed standard treatment and offered usual advice regarding life style modifications, medication i.e. aspirin, clopidogrel, statin, beta blocker, angiotensin converting enzyme inhibitor/ARBs, and sublingual nitrates SOS; while those in the Test group were given similar treatment but exposed to an intensive health education package to promote compliance. Hypertensive patients received anti hypertensive drugs and the drug dose changed during the follow up as required. At three months, CHD risk factors were assessed (except biochemical measurements) amongst both study and control groups to find out any changes. The study subjects were followed for a total of six months when all the six risk factors were assessed (including the biochemical tests) to find out reduction, if any, in the risk factors prevalence. Mortality and subsequent CHD episodes in the two groups were also recorded.

At the completion of the study, i.e. after six months of follow up, the booklet was also provided to the control group.

Follow up: The address, landline and mobile number, and email (if available) of study subjects were recorded and patients were given an exact date to attend the clinic after three and six months. The landline and mobile phone number of the investigator and translator were also given to the patients. If the subjects did not show up on the stipulated date, the investigator or the translator contacted the patient through telephone. If the patient did not show up despite the phone call, the translator called on the patient in the evening and motivated him/her to attend the clinic the next day for follow up. If the patient refused, it was labeled as lost to follow up. Patients who could not be found were also regarded as lost to follow up. The investigator also recorded mortality and subsequent episodes of CHD.

Outcome: The endpoint was reduction in the prevalence of CHD risk factors from baseline to three months and six months.

The research protocol was approved by the Institutional Ethics Committee of UCMS and GTB Hospital Delhi. The RCT was registered with Indian Council of Medical Research, New Delhi, with Registration number 2012/02/003263. Informed consent was obtained from all participants.

### 2.2 Data Collection and Measurement

The questionnaire consisted of a) socio-demographic profile b) detailed assessment of risk factors c) anthropometric measurements d) physical activity e) clinical and laboratory findings.

The researcher-administrated questionnaire was completed by all participants at baseline (two to three days before the discharge from hospital) and again at three and six months.

Weight, height, waist and hip circumference, blood pressure, and laboratory investigations including fasting and post-prandial blood sugar, HbA1c, total serum cholesterol, triglycerides, LDL and HDL were measured.

Hypertension was diagnosed based on either blood pressure ≥140/90 mmHg or isolated systolic hypertension or a known hypertensive state (Joint National Committee-7/JNC-VII, 2003).

Diabetes was defined as those who were known diabetics, and subjects with symptoms of diabetes plus random plasma glucose of ≥ 200 mg/dl and/or fasting plasma glucose ≥ 126 mg/dl and/or 2 hour post 75 g glucose ≥ 200 mg/dl. HbA1C more than 7% indicated poor glycemic control whereas HbA1C equal to or less than 7% showed satisfactory control of diabetes (ICMR, 2005). Either of these two criteria was used for control of diabetes. Above-mentioned glucose levels were considered uncontrolled diabetes. Patients with impaired fasting glucose (IFG) and impaired glucose tolerance (IGT) were not considered as diabetic. Hypertension and diabetes mellitus are known CHD risk factors. Once diabetes and hypertension develop, they cannot be cured. Therefore, there will be no reduction in their prevalence even after intervention. Hence, reduction in these two risk factors means their adequate control.

Obesity: Based on Asian Indian-specific cut-offs, a BMI>25 kg/m^2^, and/or waist circumference ≥90 cm in men and ≥ 80cm in women are taken as CHD risk factors. Either definition was used for obesity(Misra et al., 2009).

Tobacco: Current tobacco smoking in any form (bidis, cigarettes, cigars, pipes or any other smoke products) included daily and/or occasional smoking. Use of non smoke tobacco was also enquired. Tobacco use was defined as smoking tobacco, chewing gutkha, or consuming pan masala (containing tobacco).

Lipid profile disorders: Dyslipidemia was defined according to the USA National Cholesterol Education Program (NCEP) criteria (high LDL cholesterol ≥100 mg/dl and low HDL cholesterol as < 40 mg/dl in males and < 50 mg/dl in females). The cut off value was 200 mg/dl for hypercholesterolemia and 150 mg/dl for hypertriglyceridemia. Either of above mentioned criteria was used for lipid profile disorders (NCEP, 2002).

Physical inactivity: Physical Activity Level (PAL) was calculated using the proforma validated by Bharathi et al. (2000); accordingly, an individual was classified as physically inactive if the PAL was ≤ 1.55.

### 2.3 Analysis

The data collected through the questionnaires, clinical examination, and investigations was entered first into MS Excel and then to SPSS 16. Appropriate tests of significance, i.e chi-square, independent t test, etc., were applied for univariate analysis.

Generalized Estimation Equations (GEE) with logit link function was applied taking each risk factor at three time points as a dependent variable, group and time (two dummy variables) and other potential covariates as independent variables. The results were presented in odds ratios for easy interpretation. GEE was applied to find out the extent of change, if any, between the prevalence of risk factors among study and control groups over time, interaction between group and time was also included into the model to assess the change between the study and control group at 3 and 6 months. Goodness of fitness was checked for all risk factors by drawing the residual plot. The model was found adequate as observations were equally distributed below and above zero. The working correlation was decided by the comparing the Akaike information criterion.

GEE was done on all 640 subjects as GEE takes missing data (for subjects lost to follow up at three and six months) as values missing completely at random. Odds ratios were calculated by taking exponential of the estimate. We also preformed Intention to Treat (ITT) by taking Last Observation Carried Forward (LOCF) for data missing at three and six months.

Subgroup analysis was done for the gender because gender was not comparable between study and control group at baseline and the difference between the two groups was statistically significant. Besides gender, subgroup analysis was also done for different age groups as the investigator felt that there may be different risk factor profile change in patients ≤ 45 years.

The study was carried out following almost all steps as per CONSORT 2010 guidelines (CONsolidated Standards of Reporting Trials) and informed consent was obtained in each case (Rennie, 2001).

## 3. Results

Of 1430 CHD subjects admitted to CCU during January 2008 to April 2011, 790 patients were not eligible for the study (Figure 1).

GEE was used to analyze 320 subjects in the study group and 320 subjects in the control group. There was a considerable preponderance of male subjects (75.9%) while females comprised only 24.1% of the subjects. Of all, 45% and 35% of the subjects belonged to middle and high socio economic class in the study and control group, respectively. The study and control groups were fairly comparable (p>0.05) for all sociodemographic variables and clinical findings at baseline except for gender (p<0.05) (Table 1).

**Table 1:** Baseline characteristics of socio-demographic profile and clinical findings of subjects in study and control groups

Socio-demographic & clinical findings variables	Study group		Control group		df	P-value
	
	Number	Percent	Number	Percent		
Age(Years)≤40	64	20	47	14.7	1	0.076
Median	50		52			
Range	25-65		27-65			
Male	263	82.2	226	70.6	1	0.001
Urban	271	84.7	279	87.2	1	0.363
Hindu	233	72.8	227	70.9	1	0.597
Married	284	88.8	268	83.8	1	0.066
Family income (Rs.P.M.)						
Mean	9666±106		8235±902		0.072	
Median	6000		5750		0.141	
Range	2000-80000		1000-75000			
Vegetarian	180	56.2	165	51.6	1	0.234
Positive family history	51	15.9	45	14.1	1	0.507
ST elevation MI	233	72.8	242	75.6	2	0.716
Presence of chest pain	309	96.6	309	96.6	1	1.00

In the study group, 61.2% of the subjects were tobacco users, 37.8% were hypertensives, 25.6% suffered from diabetes, 66.6% lacked physical activity, 87.8% had lipid profile disorders, and 34.4% were obese at baseline whereas in the control group, 60.3% were tobacco users, 38.1% were hypertensive, 35% were obese, 71.6% lacked physical activity, 26.2% were diabetic, and 84.7% suffered from lipid profile disorders. The prevalence of all the six risk factors in the study and control group was similar with no significant difference (p>0.05). Since we collected information on various variables, we present the mean and standard deviation of these variables in study and control groups (Table 2). According to Table 2, the mean values were similar in the study and control group with no significant difference (p>0.05).

**Table 2:** Comparison of mean values of variables related to risk factors in subjects in study and control groups at baseline

Variables		Study group	Control group	p-value

		Mean ± SD	Mean ± SD	
Body Mass Index		23.8 ± 3.9	23.5 ± 4.3	0.470
Waist Circumference	Male	90.3 ± 10.7	89.5 ± 9.0	0.398
	Female	91.3± 10.4	92 ± 11.3	0.728
Hip Circumference	Male	89.4 ± 7.3	88.2 ±7.1	0.064
	Female	91.3 ± 8.6	91.6 ± 8.9	0.853
Systolic Blood Pressure		128.1 ± 25.1	131.3 ± 28.4	0.126
Diastolic Blood Pressure		81.1 ± 14.5	82.5 ± 14.2	0.203
Fasting Blood Sugar		108.8 ± 36.4	110.4 ± 42.6	0.609
2-hour Post Prandial Blood Glucose		161.8± 65.8	161.7 ± 64.3	0.993
HbA1C		10.7 ± 1.6	10.6 ± 1.6	0.706
Serum cholesterol		158 ± 42.1	160.4 ± 46.6	0.488
Serum triglycerides		137 ± 71.1	132.1 ± 69.2	0.388
HDL-Cholesterol		35.3 ± 8.1	37 ± 8.4	0.060
LDL-Cholesterol		95 ± 35.8	98 ± 40.4	0.277
Physical Activity Level		1.3 ± 0.1	1.3 ± 0.1	0.647

BMI: Body Mass Index; WC: Waist circumference; HC: Hip Circumference; SBP: Systolic Blood pressure; DBP: Diastolic Blood Pressure; FBS: Fasting Blood Sugar;2-hPG: 2 hours post prandial blood glucose; PAL: Physical Activity Level.

The subjects in our RCT (in both study and control group) comprised patients with the first episode of CHD. As we followed these patients, we also enquired about subsequent episodes of CHD. Seven and five patients suffered the second episode of CHD in the study and the control group respectively in the first three months of follow-up. In the subsequent follow-up from 3-6 months, 3 more patients in the study group and 6 more patients in control group experienced a second episode of CHD. There was no significant difference in the occurrence of the second episode of CHD between study and control groups (p=0.256). None of the patients in study and control groups suffered more than two episodes of CHD. Thus, in total, 10 patients in the study group and 11 patients in the control group suffered subsequent episodes of CHD. Among patients with the second episode of CHD, 5 patients in the study group and 3 patients in the control group died in the first three months of follow-up. In the subsequent follow-up from 3-6 months, 4 patients in the study group and 5 patients in the control group died. There was no significant difference in mortality due to CHD between study and control groups (p=0.475).

GEE models was applied for each of risk factor separately because risk factors are correlated with each other and time (two dummy variables), group and interaction between group and time along with other variables such as age, sex, diet, religion, family history of CHD, SES (lower middle, upper lower + lower, and upper + upper middle, categories were collapsed due to get adequate number) were included in the GEE model. Interaction between the time and group is found to be significant at 6 months for hypertension, tobacco, physical activity, and obesity and at 3 months only hypertension is significant, whereas interaction for lipid profile disorder, diabetes is insignificant at 6 months. Significant interaction represents the change in risk from baseline is significant at respective follow-up months between the study and case group. The working correlation was decided by the comparing the QIC, lowest score provide best correlation.

**Within group reduction:** Since interaction between group and time was significant, within group changes of each risk factors was compared by fitted the GEE models separately in study and control group taking time and other variables into the models, results were shown in Table 3.

**Table 3:** Effect of lifestyle intervention on risk factors in subjects in study and control groups

Risk factors	Baseline (Ref)	Study group						Control group					
	
		Three months			Six months			Three months			Six months		
	
		OR	95% CI of OR		OR	95% CI of OR		OR	95% CI of OR		OR	95% CI of OR	
Hypertension	1	0.41	0.289	0.586	0.30	0.193	0.467	1.08	0.805	1.445	1.05	0.775	1.408
Obesity	1	0.77	0.655	0.910	0.58	0.452	0.736	0.87	0.741	1.026	0.98	0.796	1.214
Tobacco	1	0.14	0.100	0.194	0.08	0.057	0.123	0.24	0.180	0.327	0.18	0.128	0.245
Lipid profile disorders	1	----	-----	----	0.67	0.448	1.088	-----	----	----	1.21	0.781	1.842
Lack of physical activity	1	0.97	0.790	1.186	0.63	0.515	0.763	1.27	1.024	1.570	1.20	0.964	1.535
Diabetes	1	----	-----	----	0.52	0.383	0.701	----	-----	----	0.71	0.537	0.939

In study group, there was a significant reduction in hypertension (P<0.001), tobacco use (P<0.001), and obesity (p=0.002 and P<0.001) at three and at six months, whereas there was no significant reduction in lack of physical activity at three months (P=0.754) while Physical activity reduced significantly at six months (P<0.001) in the study group. There was a no significant reduction in lipid profile disorders at six months (P=0.112), whereas diabetes showed significant reduction at six months (P<0.001). In control group, there was significant reduction in take of tobacco at both 3 months (P<0.001) and 6 months (P<0.001) from baseline and diabetes at 6 months, while diabetes reduction is also significant at 6 months (P=0.016). There is no change in other risk factors.

**Change comparison between groups**: There was a significant change between study group as compared to control group at 6 months for hypertension, obesity, physical activity, and tobacco whereas in lipid profile and diabetes change was insignificant. However, there is significant change was found only in hypertension at 3 months (Table 4).

**Table 4:** Comparison the proportion of risk factor in case and control groups

Risk factor	P-value at 3 months	P-value at 6 months
HTN	0.000 <0.001	0.000 <0.001
Physical activity	0.006	0.000 <0.001
Tobacco	0.023	0.003
Obesity	0.449	0.008
Lipid Profile	------	0.378
Diabetes	------	0.146

The results of ITT showed no significant difference as compared with GEE.

There was a statistically significant difference in male and female composition in study and control groups at baseline (p=0.001). Therefore, gender was entered as a covariate in GEE and its effect was studied for all six risk factors in both groups. There was no statistically significant interaction between gender and group (study and control).

In the present study, 150 (23.4%) subjects aged less than 45 years, i.e they had premature CHD. We analyzed the association of age with group (study and control) for all six risk factors but found no significant interaction.

## 4. Discussion

In India, there is hardly any hospital based study showing the benefits of lifestyle modification on coronary risk factors. The present study showed promising results about the effect of lifestyle intervention in reduction of CHD risk factors in a tertiary care hospital in East Delhi. Some earlier studies from India with small sample sizes have shown the beneficial effects of yoga on the progression and risk factors of CHD (Bijlani et al., 2005; Yogendra et al., 2004). Compared to these studies, our study had a much larger sample size of 640 patients with a lot of scientific vigor and a much longer follow-up. Other studies on the effect of lifestyle modification based on yoga have only discussed its effects on body weight, blood pressure, blood chemistry, etc. in non CHD patients (Kumar, Sukh, Jagbir, & Singh, 2008).

Our study showed a significant reduction in hypertension at three and six months in the study group as compared to the control group.

Periasamy et al. (2012) in Coimbatore showed the impact of a pre formatted program of lifestyle modification in patients with angiographically proven CAD. The authors reported a significant reduction in blood pressure in these patients. Results similar to our study regarding the decline in hypertension in the study group as compared to the control group have also been reported by other researchers from around the world (Aslanabadi et al., 2008; Chainani-Wu et al., 2011; Sartorio et al., 2001; Srimahachota et al., 2010).

There was a statistically significant reduction in tobacco use at three and six months in the study group as compared to the control group. Periasamy et al showed the impact of lifestyle modification on the clinical and biochemical profile of 136 CAD patients and showed a significant reduction in tobacco use (2012).

Aslanabadi et al. (2008) showed that before implementing a lifestyle modification program, 30% of the patients in the intervention group were smokers while only 10% were still smoking at the end of the follow-up period of two years in comparison with the 30% smokers in the usual care group at the end of the follow-up. They observed a significant reduction in smoking in the intervention group (p<0.05). In a study in Vestfold (Norway), 50% of the patients in the intervention program group and 42% in the usual care group were smokers at baseline. At six months, 55% of the smokers in the intervention group and 33% in the usual care group stopped smoking (p<0.05) (Ottestad, 2003).

Our study showed a decline in lipid profile disorders at six months in the study group as compared to baseline whereas an increase was observed in lipid profile disorders at six months compared to baseline in the control group although the difference was not significant. There was a significant reduction in lipids at six months in the study group as compared to the control group.

Manchanda et al. (2000) showed a significant reduction in serum total cholesterol, LDL and triglyceride in the intervention group as compared to the control group. Likewise, Yogendra et al. (2004) found statistically significant changes in the serum total cholesterol and LDL in their interventional study. In another study on a heterogeneous group of patients with hypertension, CAD, DM, and a variety of other illnesses using comprehensive lifestyle education program based on yoga, Bijlani et al. (2005) reported that fasting plasma glucose, serum total cholesterol, LDL, VLDL, the ratio of total cholesterol to HDL and triglycerides were significantly lower and HDL was significantly higher on the last day of the lifestyle educational course compared to the first day of the course.

Since hypercholesterolemia, especially high LDL, leads to the progression of atherosclerosis and adversely affects the prognosis of CHD, it warrants urgent modification. Therefore, the reduction of LDL in the study group is noteworthy. Similar findings have been published by other authors in various countries (Aldana et al., 2008; Haskell et al., 1994; Ketola, Makela, & Klockars, 2001; Ornish et al., 1998; Pugliese et al., 2007).

Another significant finding of our study was the effect of lifestyle modification on reduction of the prevalence of obesity in study group as compared to the control group at six months. Obesity reduced significantly at three months and six months in the study group as compared to baseline. In the control group, obesity slightly increased from three months to six months as compared to baseline but the difference was insignificant. Likewise, Periasamy et al reported a reduction in body weight following lifestyle modification in CHD patients (2012). The benefit of intensive lifestyle modification on weight reduction lasted even up to five years (only in the interventional group) in the study by Ornish et al. (1998) in CHD patients. A study by Chainani-Wu et al. (2011) demonstrated a significant decrease from baseline in both weight and BMI in CHD patients and high risk groups. In Italy, a body weight reduction as much as 4.1% was reported by Sartario et al. (2001) in a short term non-pharmacological intervention program.

Our study showed a significant increase in physical activity in the study group as compared to the control group at six months. Physical activity increased insignificantly at three months and significantly at six months when compared to baseline in the study group. In the control group, physical activity even decreased further at three months which was significant. At six months, physical activity increased insignificantly as compared to baseline.

The studies conducted by Chainani-Wu et al. (2011) and Pugliese et al. (2007) demonstrated that there was a significant reduction in sedentary lifestyle in the lifestyle intervention program group as compared to conventional treatment group. Some other studies have also reported increased exercise capacity only in the interventional group as compared to the control group (Haskell, et al., 1994; Niebauer et al., 1997).

There was no statistically significant difference in the occurrence of the second episode of CHD and mortality between the study and control groups. Contrary results have been reported by other researchers whereby they have published findings depicting a significant reduction in either the subsequent episode of CHD alone or together with the reduction in mortality. This variation between our and their results is likely due to the fact that while our follow-up period was only six months, these studies had much larger follow up periods ranging from three to six years (Bolinder, Alfredsson, Englund, & de Faire, 1994; Suzuki, Kohro, Hayashi, Yamazaki, & Nagai, 2012).

CHD is much more common in males as compared to females and recently the advancing epidemic of CHD has affected a large number of young Indians. We analyzed the results for gender and in different age groups to see if they behaved differently after lifestyle modification in study and control groups. As described in the results, gender and age sub classifications did not yield any different results in either male or female CHD patients or in younger/older CHD patients meaning that lifestyle modification in the study and control groups were affected uniformly across gender and age group in both study and control groups. Our study applied the intervention package to the study patients which was an exhaustive one, consisting of repeated health motivational messages, closer monitoring, more effective defaulter retrieval mechanisms etc. which requires very careful planning and management in preventive cardiology clinics in non-study conditions. This study was done in East Delhi in CCU and PCC for three years. However, some caution is needed whether these results can be replicated in over crowed cardiology clinics. We feel that when patients turn-up for their follow-ups in Preventive Cardiology Clinics e.g. when they have to wait for consultants in long queues, this shortcoming can be turned into opportunity by showing them educational packages and motivating them to change their life styles and come for follow-ups regularly. However, more studies are needed to validate the results of our study findings in PCC clinics across the country by involving cardiologists in them. Most PCCs in developing countries lack sufficient staff and financial resources for these activities and therefore there is a need for recruitment of such para-medical staff and additional budget.

## 5. Limitations

We measured lipid and glucose levels only at the time of admission and at six months. We could not perform these biochemical investigations at three months. The diagnosis of CHD in the present study was based on Monica criteria. For feasibility reasons, we could not conduct quantitative coronary angiography which is the gold standard. We did not enquire about the complications of CHD like arrhythmia, CVA, etc. during the follow-up period. We assessed risk factors modification for only six months. Whether patients would maintain these lifestyle modifications beyond six months or even years remains unanswered. The follow-up time was fairly short to assess any real modification in the risk factors. Hypertensive patients were given anti hypertensive drugs and dose of the drug was changed during the follow up as required, which is a confounding factor in assessing the real impact of lifestyle intervention on blood pressure in our study and merits further in-depth studies addressing this issue. We included only new CHD patients aged 25-65 years. Therefore, our result may be generalized only to this age group and those with the first episode of CHD, and not to patients with the second or third episodes of CHD.
